# Genetic Creutzfeldt-Jakob disease linked to the E200K mutation: a large cohort study

**DOI:** 10.1007/s00401-026-02975-x

**Published:** 2026-01-13

**Authors:** Brian S. Appleby, Matteo Manca, Megan S. Piazza, Travis D. Kerr, Antonio Cornacchia, Alberto Bizzi, Allison Kraus, Mark L. Cohen, Ignazio Cali

**Affiliations:** 1 Department of Pathology, School of Medicine, Case Western Reserve University, Cleveland, USA; 2 Department of Neurology, School of Medicine, Case Western Reserve University, Cleveland, USA; 3 Department of Psychiatry, School of Medicine, Case Western Reserve University, Cleveland, USA; 4 National Prion Disease Pathology Surveillance Center, Cleveland, OH 44106 USA; 5 Center for Human Genetics Laboratory, School of Medicine, Case Western Reserve University, Cleveland, USA; 6 University Hospitals Cleveland Medical Center, Cleveland, USA; 7 Center for Immunotherapy and Precision Immuno-Oncology, Lerner Research Institute, Cleveland Clinic, Cleveland, OH USA; 8 UOC Neuroradiologia, Fondazione IRCCS Istituto Neurologico Carlo Besta, 20133 Milan, Italy; 9 School of Medicine and Surgery, Kore University, 94100 Enna, Italy

**Keywords:** Creutzfeldt-Jakob disease, E200K, Prion protein, Codon 129, RT-QuIC, Lesion profile

## Abstract

Creutzfeldt-Jakob disease (CJD), the most common human prion disease, is an invariably fatal neurodegenerative disorder affecting 1.5 cases per million individuals per year. About 10–15% of the human prion diseases are caused by a pathogenic variant in the prion protein (PrP) gene (*PRNP*), and the most common genetic human prion disease is CJD (gCJD) linked to a glutamic acid to lysine substitution at codon 200 (E200K) of *PRNP*. The polymorphic codon 129 methionine (M)/valine (V) genotype has a strong effect on disease phenotype. In the present study, we retrospectively evaluated many features of gCJD E200K cases with respect to the 129MV polymorphism, type of scrapie prion protein (PrP^Sc^), demographic, clinical, laboratory, histopathology, and molecular features, including western blot examination and real-time quaking-induced conversion assay. Analyses were also performed to determine statistically significant features between E200K haplotypes (e.g., codon 129 genotype in *cis* with the mutated allele) and codon 129 genotypes. This study found that codon 129 polymorphism affects several disease features of gCJD E200K. Specifically, histopathologic differences were found between patients with different 129 haplotypes and genotypes. We have identified five groups or subtypes of E200K associated with either PrP^Sc^ type 1 or 2. Other E200K cases showed mixed (i) PrP^Sc^ types or (ii) pathological features of 129 M and 129 V haplotypes. To our knowledge, this study describes the largest cohort of 177 E200K cases and provides new insight into the wide range of phenotypes associated with this common CJD genetic variant.

**Supplementary Information:**

The online version contains supplementary material available at 10.1007/s00401-026-02975-x.

## Introduction

Prion diseases are a group of invariably fatal, transmissible, neurodegenerative disorders affecting humans and other mammals [13, 18]. The central pathogenic event in prion disease is the conversion of the normal prion protein (PrP^C^) into the abnormal scrapie PrP (PrP^Sc^), and its accumulation in the brain [48]. The PrP^C^ to PrP^Sc^ conformational change is characterized by a refolding of major α-helices motifs of PrP^C^ into β-sheet conformers that are prone to aggregate into amyloidogenic fibrils. Once seeds of PrP^Sc^ are formed (with PrP^Sc^ driving PrP^C^ → PrP^Sc^ conversion) and reach a critical threshold concentration, the proteopathic process of PrP^Sc^ formation is thermodynamically favored and becomes autocatalytic [54]. Prion disease may occur via genetic, acquired, or sporadic etiologies [5]. In addition to disease etiology, prion diseases are phenotypically heterogeneous disorders, with phenotypic variability stemming from a multitude of possible strain-specific conformations of PrP^Sc^ [19]. Prion strains and their related disease phenotypes are faithfully, and serially, propagated to syngeneic hosts [3, 8, 56]. While the worldwide yearly incidence of prion disease is  ~ 1–2 new cases per million individuals, the genetic form is estimated to represent approximately ~ 10–15% of the human prion diseases [1, 28, 50]. Genetic human prion diseases are linked to pathogenic genetic variants in the PrP gene (*PRNP*), with up to 60 known variants [28]. Each pathogenic variant is associated with distinctive histopathological features and clinical profiles in the affected patients with some variability. Furthermore, genetic prion diseases can be classified into three major disease phenotypes that include (1) genetic Creutzfeldt-Jakob disease (gCJD), (2) Gerstmann-Sträussler-Scheinker (GSS), and (3) fatal familial insomnia (FFI) [1].

The most common pathogenic mutation in the *PRNP* gene is the glutamic acid (Glu, E) to lysine (Lys, K) substitution at codon 200 (E200K) leading to gCJD E200K (hereafter indicated as E200K) [2, 10, 21, 32]. Other mutations at codon 200 include the rare E to glycine (G) and E to aspartic acid (D, Asp) substitutions (i.e., E200G and E200D, respectively) [25, 27].

In general, the clinical features of E200K are similar to those of sporadic CJD (sCJD), and are characterized by progressive dementia and ataxia with mean age at symptom onset of ~ 61 years-of-age [2, 28, 38]. Disease duration of E200K can range from ~ 2 to 41 months [30]. Brain magnetic resonance imaging (MRI) in E200K tends to demonstrate hyperintense regions in the cortex and/or basal ganglia on diffusion weighted imaging and electroencephalography findings of periodic sharp-wave complexes, both suggestive of CJD [34]. Concentrations of cerebral spinal fluid (CSF) markers, such as 14–3-3 and total tau proteins are elevated [34]. Furthermore, detection of PrP^Sc^ by CSF real-time quaking-induced conversion (RT-QuIC) has become a gold standard for a *pre*-*mortem* diagnosis. RT-QuIC shows distinct seeding activity with an overall sensitivity and specificity of 90.3% and 98.5%, respectively, that varies between human prion strains [49]. Cerebral spinal fluid-PrP^Sc^ from patients with E200K has shown to be amplified by RT-QuIC assay with 100% sensitivity [2, 42, 49].

Similar to sCJD, two types of PrP^Sc^ have been described in E200K. Type 1 (T1) PrP^Sc^ features a major unglycosylated band migrating to ~ 21 on gel, while type 2 (T2) unglycosylated PrP^Sc^ migrates to ~ 19 kDa [2, 27]. Furthermore, the co-occurrence of PrP^Sc^ T1 and T2 has been described in E200K, but at lower rates than in sCJD [2]. Notably and like other human prion disease, the PrP^Sc^ type modulates disease phenotype in E200K [2, 27]. In addition to causative variants in *PRNP* and PrP^Sc^ type, the methionine (M)/valine (V) polymorphism at codon 129 has a strong influence on the disease phenotype. This alteration affects susceptibility to, duration of, clinical presentation, and neuropathological features of the disease [2, 22, 44, 52, 58]. The 129 M/V polymorphism also affects the development of prion disease strains [11]. For example, when D to asparagine (N) substitution at codon 178 (D178N variant) is in *cis* with the 129 M allele (D178N-129 M haplotype), the resulting disease phenotype is FFI. On the contrary, when the mutation is in *cis* with the 129 V allele (D178N-129 V haplotype), the disease phenotype overlaps with CJD in several aspects [41]. Similarly, in Gerstmann-Sträussler-Scheinker disease (GSS) when proline (P) to leucine substitution at codon 102 (P102L variant) is in *cis* with the 129 M allele, the disease course is shorter and the clinical presentation differs from those of patients with the P102L variant in *cis* with the 129 V allele [23, 60]. The exact mechanism by which the polymorphic codon alters disease features remains to be fully elucidated. In a recent model, it has been shown that the preferential conversion of PrP^C^-129 M or PrP^C^-129 V to PrP^Sc^ is associated with different disease phenotypes [13, 43].

In the general population, the 129MM and 129VV genotypes range from 6 to 94% and 0–47%, respectively, depending on the geographical region [15, 26, 29, 53]. Due to the predominance of the 129 M haplotype, the majority of E200K cases described in the literature are in *cis* with codon 129 M [2, 16, 20, 32]. There is little information regarding the clinical and histopathologic features of E200K of the 129 V haplotype, with few 129VV cases (always associated with T2) reported worldwide [2, 24, 32].

Recently, it has been shown that E200K can be divided into 4 subtypes, with 3 subtypes sharing sCJD phenotypic features. These subtypes include: (1) MM(MV)1 or M1; (2) MV2/VV2 or V2 (K) with or without kuru (K) plaques; (3) MM(MV)2C or M2C; and (4) MV with type “intermediate” or M “i” (the latter subtype is not recognized in sCJD)[2]. An additional E200K subtype may be associated with thalamic dysfunction and insomnia at onset [16, 55, 59].

In the present report, we present the demographic, clinical, neuropathological, and molecular features of 177 individuals carrying the E200K *PRNP* mutation in *cis* with the 129 M or 129 V (identified as 129 M and 129 V haplotypes, respectively). We describe five phenotypically distinct E200K subtypes. A common subtype is the MM(MV)1, which represents approximately 70% of E200K “pure” subtypes, while the MM(MV)2, FI (a subtype mimicking fatal insomnia), MV2 and VV2 are less common, but readily identifiable. Like several other human prion diseases, the distribution of PrP^Sc^ types, clinical phenomenology, and histopathological characteristics are strongly influenced by the codon 129 genotype in E200K. This comprehensive study provides a detailed phenotypic and molecular characterization of the largest E200K cohort.

## Materials and methods

### Case study

181 E200K cases were obtained from the National Prion Disease Pathology Surveillance Center (NPDPSC) through queries of the brain bank database for this mutation. Four cases did not have full haplotype data and were excluded from these analyses. The subsequent 177 cases included in the study were divided into four groups based upon the presence of (i) PrP-codon 129 M or 129 V haplotypes, and (ii) M or V in the normal allele (i.e., E200K 129MM, -MV, -MV, -VV genotypes) (Fig. S1). The largest group of E200K cases belonged to the 129MM genotype (n = 116) and was followed by the -MV (n = 40), -MV (n = 12), and -VV (n = 9) (Fig. S1). Results on the type of the proteinase K (PK)-resistant PrP^Sc^ were not available in three E200K cases with 129MM genotype.

Available data allowed comparative data analyses of age at death and disease duration in 175 and 167 cases, respectively. Cases were also stratified by PrP^Sc^ type. Specifically, the presence of either PrP^Sc^ T1 or T2, or their co-occurrence, was determined by 3F4 antibody of three (70%; frontal and occipital cortices, and cerebellum), two (21%; typically, frontal or occipital cortex and cerebellum), or one (variable) brain region (9%) under the following experimental conditions: (1) tissue homogenization with 1X LB100 pH 8.0 (see below) and the use of 15% Tris–HCl (8.7 cm-long) precast gels (see below). In addition, we repeated the western blot of 18 E200K and relative controls (e.g., 8 sCJD, and one FFI cases). E200K cases included: two MM1 (cases 2 and 5; Table S1), three FI (cases 6–8), one MM2 (case 9), and 2 each of MV1 (cases 11 and 12), MV2 (26 and 27), MV2 (31 and 32) and VV2 (cases 34 and 37). Sporadic CJD cases included three MM1, and one each of MV1, MM2, MV2K and MV2C, and VV2. Classification into molecular subtypes was done according to Parchi et al. classification system [47].

### Histopathology and lesion profiles

To assess the histopathological phenotype, we performed pathological examinations of 60 E200K cases encompassing all genotypes at codon 129. Thirty-seven of the 60 cases, representative of various subtypes, were studied in more detail (Table S1). These cases included: (i) 9 -MM, (ii) 18 -MV, (iii) 5 -MV, and (iv) 5 -VV. Pathology reports available in 90 additional E200K cases were reviewed. Histology and PrP immunohistochemistry was carried out as previously described [9]. Thirty of the 37 E200K cases were suitable for a semi-quantitative evaluation of the severity and distribution of pathological lesions (spongiosis and gliosis lesion profiles). Severity of the lesions were determined in ten brain regions including the frontal, temporal, parietal, occipital, and entorhinal cortices (FC, TC, PC, OC, EC), hippocampus (HI), basal ganglia (BG), anterior thalamus (TH), substantia nigra (SN), and cerebellum (CE). Severity of spongiform degeneration (SD) was scored on a 0–4 scale (0: absent; 1: mild; 2: moderate; 3: severe; 4: status spongiosus), whereas severity of gliosis and neuronal loss severity was scored on a 0–3 scale (0: absent; 1: mild; 2: moderate; 3: severe) [8, 9]. Each point of the lesion profile was expressed as mean ± standard error of the mean (SEM) of SD and gliosis in all brain regions except for the cerebellum, which included scoring of granule cell loss. Finally, thalamic neuronal depopulation on a 0–3 score was assessed in E200K linked to the MM2 and FI subtypes.

### Brain homogenate preparation and western blot analysis

The frontal cortex of all E200K, sCJD, and FFI cases was used for typing purposes. Brain homogenates (20% wt/vol) were prepared with 1X Dulbecco's phosphate buffered saline and then mixed with 2X lysis buffer with 100 mM Tris (1X LB100: 100 mM NaCl, 0.5% Nonidet P-40, 0.5% sodium deoxycholate, 10 mM EDTA, 100 mM Tris–HCl, pH 6.9). Supernatants (S1) were collected after centrifugation at 1,000 × g for 5 min. For characterization of the PrP^Sc^ of three E200K-MM2 cases with a histopathological phenotype (histotype) mimicking fatal insomnia, and in one case with FFI, S1 were spun at 100,000 × g (1 h at 4 °C), and pellets (P2) were concentrated tenfold and sonicated. S1 and P2 samples were digested with 10 and 5 Units (U)/ml PK, respectively (1 U/ml is equal to ~ 20 μg/ml PK), at 37 °C for 1 h. Enzymatic reaction was stopped by the addition of 3 mM phenylmethylsulfonyl fluoride. Samples were mixed (1:1) with 2X Laemmli buffer (6% SDS, 20% glycerol, 4 mM EDTA, 5% β –mercaptoethanol, 125 mM Tris–HCl, pH 6.8) and then denatured (10 min at 100 °C). Western blots were performed as previously described [8]. For typing of PrP^Sc^, proteins were separated using 15% Criterion™ Tris–HCl Precast Gels (W x L: 13.3 cm × 8.7 cm), which were then blotted onto the Immobilon-FL PVDF membrane. Membranes were blocked with Odysseys blocking buffer, co-incubated with primary antibodies 3F4 (1: 10,000) and Tohoku-2 (1: 10,000). Following washing in 0.1% Tween-20 in Dulbecco phosphate buffer saline, membranes were incubated with Abs IRDye 800CW goat anti-mouse IgG (1: 10,000) or IRDye 680RD goat anti-rabbit IgG (1: 10,000). PrP^Sc^ was visualized using the Odyssey infrared imaging system (LI-COR Biosciences).

### Real-time quaking-induced conversion assessments in brain tissue

Frontal cortex brain tissue homogenates from representative cases of sCJD, E200K, FFI, and sFI were used for end-point dilution analysis to estimate PrP seeding doses (log_10_ SD_50_/mg tissue). RT-QuIC reaction mix was composed of 10 mM phosphate buffer (pH 7.4), 300 mM NaCl, 0.1 mg/ml truncated recombinant Syrian Golden Hamster residues 90–231, 10 μM Thioflavin T (ThT), 1 mM ethylenediaminetetraacetic acid tetrasodium salt hydrate (EDTA). A volume of 49 μl of such mix was loaded into the wells of a 384-well plate (Nunc). 20% of human brain homogenates were serially diluted in Sample Diluent Buffer (PBS, 0.1% SDS and 1X Gibco N-2 supplement) and used to seed four replicate RT-QuIC reactions (1 μl/well). The plate was then sealed with a plate sealer film (Nalgene Nunc International) and incubated at 50 °C in a BMG FLUOstar Omega plate reader with cycles of 1 min shaking (700 rpm, double orbital) and 1 min rest throughout the 30 h-incubation time. ThT fluorescence measurements (450 ± 10 nm excitation and 480 ± 10 nm emission; bottom read) were taken every 45 min. The mean and standard deviation of the negative control (non-prion disease brain) baseline across four replicate wells, excluding the first 9 reads (to allow for temperature acclimatization and establishment of a consistent baseline) were used to calculate corresponding z-score values for each read. Replicate wells whose z-score value exceeded ± 1.96 (outside of the 95% confidence level) were excluded from threshold calculations. The threshold for a positive read was then calculated using the average of the remaining wells summed with 10 × standard deviation of the baseline. End-point dilution titrations were used to quantitate RT-QuIC prion-seeding activity by determining the sample dilution giving positive reactions in 50% of replicates as per methods of Spearman-Kärber [14], previously described [57].

### Analysis of cerebrospinal fluid 14–3-3 protein, total tau, and PrP seeding activity

Levels of CSF 14–3-3 and total tau proteins, surrogate markers for prion disease, were measured by western blot and enzyme linked immunosorbent assays (ELISA), respectively [17]. Seeding assays using the real-time quaking-induced conversion (RT-QuIC) was employed to measure seeding activity of PrP^Sc^ using second-generation RT-QuIC with truncated hamster PrP [49]. Briefly, 15 µl of neat CSF were used to seed the wells of a 96-well plate preloaded with 85 µl or reaction mix (10 mM phosphate buffer (pH 7.4), 300 mM NaCl, 0.1 mg/mL SHa rPrP(23–231), 10 μM thioflavin T (ThT), 1 mM ethylenediaminetetraacteic acid tetrasodium salt (EDTA) and 0.002% sodium dodecyl sulfate (SDS)). The plates were sealed and incubated for 60 h in FLUOstar Omega plate reader (BMG LABTECH) at 55 °C, with intermitting cycles of shaking (60 s) and rest (60 s). ThT fluorescence measurements were taken every 45 min.

### Sanger sequencing

Sequencing of *PRNP* variant and the 129MV polymorphism determination was performed. Briefly, exon two of *PRNP* was amplified by polymerase chain reaction (PCR) followed by bi-directional sequence analysis. A restriction enzyme digestion was utilized to determine the phase of the variants. The reference sequence used for *PRNP* was NM_00311.3.

### Demographic and clinical data

Demographic and clinical data were retrospectively analyzed and included the following data, if available: (1) gender, (2) race/ethnicity, (3) family history of prion disease, (4) age at death, (5) disease duration, (6) clinical symptoms/signs at onset, (7) CSF 14–3-3 protein, total tau, and RT-QuIC results, (8) brain MRI results, and (9) EEG results. Clinical symptoms/signs were stratified and included: cognitive, cerebellar, visual, myoclonus, pyramidal, extrapyramidal, seizures, sensory abnormalities, sleep disturbances, mood and anxiety changes, hallucinations, delusions, and personality or behavior changes. Determination of a positive MRI was based on 2009 clinical diagnostic criteria [61]. Brain MRI images were all reviewed by AB and BSA.

### Statistical analyses

To assess the effect of the PrP-codon 129 genotype on clinical features, multiple statistical analyses were employed. Variables were examined across full genotypes and haplotypes. Statistical analyses were done using IBM SPSS v28 and GraphPad Prism 9.0. Time related variables (age and duration) were initially evaluated via Log rank tests. One-way ANOVA and Cox proportional hazards regression models were used for calculating proportional hazard ratios (HRs) and corresponding 95% confidence intervals, to estimate the effect of polymorphism on disease duration and age at disease onset, while accounting for genotype and haplotype distributions. Pairwise comparisons after Bonferroni correction were used with two-sided p-value. Categorical variables were evaluated using Chi-square analyses (2-sided) or Fisher’s exact test if there were less than 5 cases in a category. Continuous variables were evaluated using ANOVA. A P-value < 0.05 was considered statistically significant. The Student’s t-test and one-way ANOVA were used for comparative analyses of lesion profiles and cerebellar atrophy, respectively. In RT-QuiC experiments, given the limited and unbalanced cohort sizes, the statistical comparisons of seeding doses and ThT maximum curve amplitudes between sCJD and E200K cases were performed using a non-parametric two-sided Mann–Whitney U (MWU) test in GraphPad Prism 10. Single FFI and sFI cases were excluded from the statistical analyses and are shown descriptively. The Spearman–Kärber method was used to quantify prion seeding activity by estimating the SD₅₀.

## Results

### Prevalence of 129 polymorphisms and demographics

The mutation was in the 129 M haplotype in 156 (88%) cases or 129 V haplotype in 21 (12%) cases (Fig. S1). Genotypes 129MM, -MV, -MV and -VV accounted for 65%, 23%, 7% and 5%, respectively. When cases were separated by race/ethnicity, 82% were white, 15% Hispanic or Latino, and 3% included other races/ethnicities (Table 1). Male and female were present in similar proportions (52% and 48%, respectively).

**Table 1:** Demographic, clinical presentation, biomarkers, and imaging data of E200K

Codon 129 genotype		MM	MV	MV	VV	All cases
Female, % (n = 177)		**47** (55/116)^a^	**43** (17/40)	**67** (8/12)	**56** (5)	**48** (85/177)
Race/Ethnicity, % (n = 148)	White	**81** (77/95)	**94** (31/33)	**82** (9/11)	**56** (5/9)	**82** (122/148)
	Hispanic	**16** (15/95)	**3** (1/33)	**18** (2/11)	**44** (4/9)	**15** (22/148)
	Asian	**1** (1/95)	**0** (0/33)	**0** (0/11)	**0** (0/9)	**1** (1/148)
	Other	**2** (2/95)	**3** (1/33)	**0** (0/11)	**0** (0/9)	**2** (3/148)
Age at death (years, n = 175) ^b^		60 ± 10	63 ± 9	60 ± 12	56 ± 10	61 ± 10
Disease duration (months, n = 168)^b,c^		3.7 ± 3.0	11.1 ± 9.8	7.4 ± 3.1	5.8 ± 5.3	5.7 ± 6.2
Presentation, %	Cognitive	**48** (35/73)	**48** (11/23)	**25** (2/8)	**25** (1/4)	**45** (49/108)
	Cerebellar	**19** (14/73)	**22** (5/23)	**50** (4/8)	**0** (0/4)	**21** (23/108)
	Sensory	**15** (11/73)	**17** (4/23)	**25** (2/8)	**50** (2/4)	**18** (19/108)
	Pyramidal	**11** (8/73)	**9** (2/23)	**0** (0/8)	**0** (0/4)	**9** (10/108)
	Psychiatric	**7** (5/73)	**4** (1/23)	**0** (0/8)	**25** (1/4)	**7** (7/108)
Family history of CJD, % (n = 154)		**81** (79/97)	**90** (35/39)	**100** (9/9)	**56** (5/9)	**83** (128/154)
Positive CSF 14–3-3, % (n = 108)		**74** (53/72)	**38** (8/21)	**100** (7/7)	**75** (6/8)	**69** (74/108)
Total tau [pg/ml] × 1,000 (n = 80)^b,d^		5.5 ± 5	2 ± 2.7	10.9 ± 5.8	9.5 ± 9.4	5.8 ± 5.6
Positive RT-QuIC, % (n = 71)		**98** (48/49)	**100** (10/10)	**100** (6/6)	**100** (6/6)	**99** (70/71)
Brain MRI suggestive of CJD, % (n = 37)^e^		**73** (16/22)	**64** (7/11)	**50** (1/2)	**100** (2/2)	**70** (26/37)
EEG with PSWCs, % (n = 78)		**56** (32/57)	**18** (3/17)	**50** (1/2)	**50** (1/2)	**47** (37/78)

Bold values indicate percentages

*CSF* Cerebrospinal fluid,* RT−QuIC* real−time quaking−induced conversion,* MRI* Magnetic resonance imaging,* EEG* electroencephalogram,* PSWC* periodic sharp wave complexes

^a^Cases with the feature listed/total cases examined

^b^Expressed as mean±SD

^c^P<0.001 when comparing −MM and −MV; P<0.05 when comparing −MV and –VV

^d^P<0.005 when comparing MV and MV

^e^All cases reviewed at the NPDPSC

### Clinical features

**Fig. 1:**
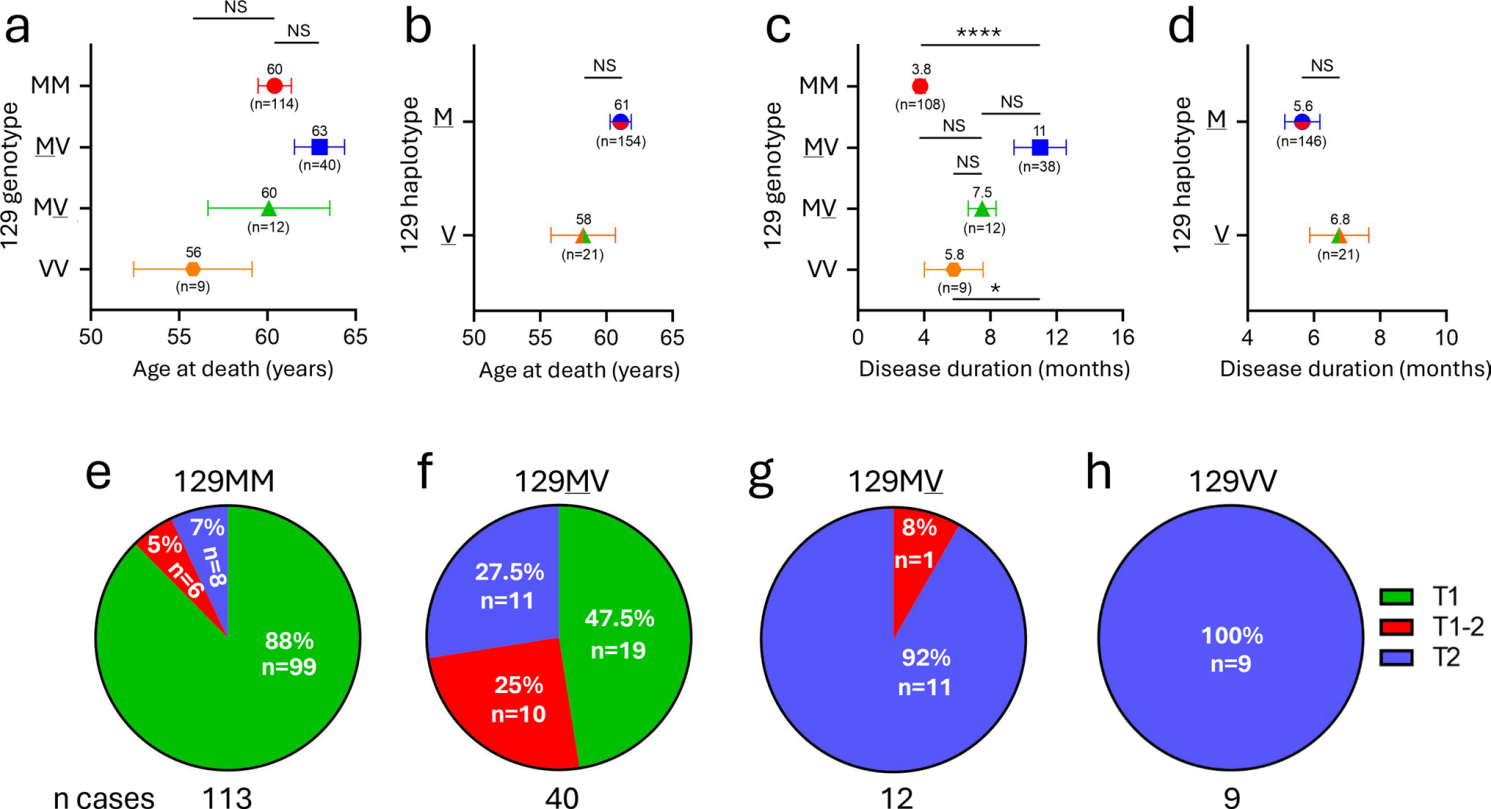
Age, disease duration and PrP^Sc^ type prevalence of the large E200K cohort. Age at death (**a**,** b**) and disease duration (**c**, **d**) across 4 groups or codon 129 variants generated by the pairing of the mutated (*cis,* M or V haplotypes) and normal (*trans,* M or V) alleles. Numbers atop symbols indicate the mean; each bar indicates standard error of the mean (SEM). ****P < 0.0001, *P = 0.01–0.05, one-way ANOVA; NS: not significant. **e**–**h** Distribution of PrP^Sc^ types (T1, T2 and mixed types or T1-2) across the four codon 129 variants. T1 prevalence is highest in -MM and absent in the V haplotype; T2 prevalence decreases in the opposite direction (-VV > -MV > -MM). T1-2 is most prevalent  in the -MV group

The mean age at death across all genotypes was 61-years-of-age and there were no statistically significant differences between codon 129 genotypes (Log Rank, P = 0.31) (Fig. 1 a and b; Table 1). Mean disease durations differed between E200K genotypes. For example, E200K-129MV cases had statistically significantly longer disease durations compared to -MM (P < 0.0001) and -VV (P < 0.05) cases (Fig. 1 c and d; Table 1). Mean age at death and disease duration did not differ significantly between -129 M and -129 V (P > 0.05) (Fig. 1 b and d). Eighty-three percent of all E200K cases reported a known family history of CJD; however, fewer codon 129 homozygotes reported a known family history of CJD compared to heterozygotes (79% vs. 92%, Fisher’s exact test, P = 0.06) (Table 1).

Most E200K cases presented with a clinical phenotype consistent with classic CJD. The plurality of cases presented with cognitive symptoms (e.g., short-term memory problems, word-finding difficulties, executive dysfunction) (45%) followed in frequency by cerebellar symptoms (e.g., ataxia, incoordination, vertigo) (21%), and sensory symptoms (e.g., paresthesia and abnormal sensations) (18%) (Table 1). There were no statistically significant differences in initial symptoms between E200K genotypes; however, presenting symptoms were associated with statistically significant differences in mean age at onset (Cox regression, Chi-square: 18.8, P < 0.005) with initial pyramidal symptoms associated with the youngest mean age at onset (54 ± 7 years) and cognitive symptoms associated with the oldest mean age at onset (64 ± 11 years).

Diagnostic test results resembled those typically observed with sporadic CJD (Table 1). Most E200K cases had positive cerebrospinal fluid (CSF) 14–3-3 results (69%) with codon 129 homozygotes having a higher percentage of positive results compared to heterozygotes (74% vs. 54%) (Chi-square: 3.92, P < 0.05). CSF total tau levels differed significantly between E200K genotypes (One-way ANOVA, P < 0.005) with -MV cases (2,065 ± 2,706 pg/mL) differing significantly from -MV (10,935 ± 5,805 pg/mL, P < 0.01) and -VV (9,508 ± 9,436 P < 0.05) cases (Table 1). All cases that underwent CSF RT-QuIC testing were positive except for one -MM case. Brain magnetic resonance imaging (MRI) was suggestive of prion disease in 70% of all cases and there were no statistically significant differences between E200K genotypes. Brain MRI images available for review demonstrated hyperintensity in the caudate and putamen on diffusion weighted imaging (DWI) and most cases demonstrated cortical involvement (70%). Periodic sharp wave complexes (PSWCs) were present on electroencephalogram in ~ 50% of all cases but were more frequent in homozygotes (56%) compared to heterozygotes (21%) (Fisher’s exact test, P < 0.01).

### E200K PrP^Sc^ type distribution across the four 129 genotypes

Similarly to several other human prion diseases, we observed a strong effect of PrP-codon 129 on the distribution of PrP^Sc^ T1 and T2. Prevalence of pure PrP^Sc^ T1 decreased proportionally across the four genotypes: it was the highest in -MM (88%), decreased by ~ 50% in -MV (47.5%), and it was absent in -MV and -VV, respectively. The prevalence of pure PrP^Sc^ T2 followed the opposite direction, as it was the only PrP^Sc^ type in -VV (100%), and decreased progressively from -MV (92%) to -MV (27.5%) to -MM, (7%) (Fig. 1e–h). Co-occurrence of PrP^Sc^ types (“type 1–2” or T1-2) affected ~ 10% of the E200K population and was more frequently observed in -MV (25%) than -MV (8%), -MM (5%), or -VV (0%) (Fig. 1e–h).

### Identification of major E200K subtypes

Following the analysis of the distribution of PrP^Sc^ type and the characterization of the histopathological phenotype, we identified five major E200K groups or subtypes, which carried either PrP^Sc^ T1 or T2. The five E200K subtypes belong to (1) -MM(MV)1, (2) -MM(MV)2, (3) -FI (indistinguishable from sporadic fatal insomnia, sFI, and FFI), (4) -MV2, and (5) -VV2 (Table 2). Furthermore, three E200K groups were characterized by co-existing PrP^Sc^ types and mixed histopathological features that were readily identified in patients with comparable proportions of PrP^Sc^ types 1 and 2 [6, 9]. On the other hand, a single histotype was observed when the ratios of co-existing PrP^Sc^ types was significantly unbalanced (i.e., T1 significantly exceeding T2 or vice versa) [6, 9]. E200K cases with co-existing PrP^Sc^ types included MM1-2 (n = 6), MV1-2 (n = 7) and MV1-2 (n = 1) (Table 2). A few E200K -129 M (MV1, n = 1; MV1-2, n = 2) had mixed M and V histotypic features (Table 2 and Table S1, cases 14, 22–24).

**Table 2:** Molecular classification and disease phenotypes of E200K

E200K subtypes	Prevalence, % (n)^a^	Age at death (years)^b^	Disease duration (months)^c^	PrP^Sc^ type	Disease phenotype
Subtypes with pure PrP^Sc^ types					
MM(MV)1	**67** (117)	61 ± 10	3 ± 2	1	Most cases presenting w. cognitive or crbl sx; diagnostic tests (including bMRI) suggest. of CJD in most cases. Fine SD often w. laminar distribution (Figs. 3a, S3 a). LP similar in MM1 and MV1 (Fig. 3l and m; Fig. S3b; Table S1). Diffuse and perineuronal PrP (Fig. 4a and b, Table S1). crbl w. plaque-like PrP occasionally affecting the granular layer (Table S1); crbl stripe-like PrP representing a common feature (Fig. 4j)
MM(MV)2^d^	**7** (12)	64 ± 10	20 ± 10	2	-MV2 (n = 11) and -MM2 (n = 1) resembling sCJDMM2; longest illness duration (20 ± 10 months); typically presenting w. cognitive or crbl sx. CSF often not suggest. of PrD. SD w. large and confluent vacuoles (Fig. 3b and c, Table S1). MV2 and -MM2 w. similar LP, but TL more severely affected in MV2 (Fig. 3n); coarse cortical pathology (Fig. 4 c and d) (Table S1). crbl (n = 2) w. stripe-like and coarse PrP (Fig. 4l and m, Table S1)
FI	**4** (6)	59 ± 9	4 ± 3	2	-MM2 cases resembling FI-histotype. Cases presenting w. cognitive or sensory sx; clinical diagnostic tests usually not suggest. of PrD (Table 1). SD virtually absent; gliosis and neuronal loss prominent in ant. thalamic and inferior olivary nuclei (Fig. 3d and j) contrasting w. lack of same feature in -MM(MV)2 (Figs. 3k and S3 c). Rare coarse deposits and crbl stripe-like PrP (Fig. 4e and n, Table S1)
MV2	**6** (11)	61 ± 12	7 ± 3	2	MV2 patients usually presenting w. crbl sx. CSF tests usually suggest. of PrD. Pathological features similar to VV2: (i) small size vacuoles, (ii) LP, presence of (iii) perineuronal and (iv) dot-like PrP (Figs. 3e and f, 4f-i, S3 d). Dot-like PrP affecting CC deep layers, less frequently subcortical nuclei. LP w. the most severe lesions and crbl atrophy; crbl medium size vacuoles and diffuse PrP
VV2	**5** (9)	56 ± 10	6 ± 5	2	VV2 patients usually presenting w. psychiatric sensory sx. CSF tests usually suggest. of PrD. Distinctive features from MV2: (i) less severe LP and crbl atrophy (Figs. 3n, S3 e and f); (ii) crbl small vacuoles (Fig. 3i) and plaque-like PrP (Fig. 4p)
Subtypes with co-existing PrP^Sc^ types					
MM1-2	**4** (6)	53 ± 11	7 ± 8	1–2	Mixed histopathology w. predominant MM1 features. Most presenting w. crbl sx. CSF 14–3-3 usually negative, elevated tau levels; bMRI usually suggest. of CJD
MV1-2	**4** (7)	64 ± 6	8 ± 5	1–2	Resembling MV1 in CC, MV2 in hippocampus., subcortical nuclei and crbl. Most presenting w. cognitive sx. CSF 14–3-3 usually negative, low tau levels; bMRI sometimes suggest. of CJD
MV1-2	** < 1** (1)	51	9	1–2	Histopathology similar to MV2. Cognitive sx at presentation
Subtypes with mixed M and V histotypes^d^					
MV1	** < 1** (1)	62	9	1	Dot-like PrP, cognitive sx at presentation. CSF 14–3-3 negative, low tau levels; bMRI not suggest. of CJD
MV1-2	**2** (3)	67 ± 6	16 ± 11	1–2	Dot-like PrP; presenting w. cogn. or sensory sx, elevated tau levels; bMRI sometimes suggest. of CJD

Bold values indicate percentages

*w.* with, *sx* symptoms, *crbl* cerebellar/cerebellum, *sx* symptoms, *PrD* prion disease, *bMRI* brain MRI, *suggest. of* suggestive of, *SD* spongiform degeneration, *LP* lesion profile, *TL* temporal lobe, *not suggest.* not suggestive, *CC* cerebral cortex

a, b, c Based on a 173, b 175, and c 167 E200K cases. d One E200K MM2 case with atypical molecular and histopathological features, and three E200K-129MM are not included

### PrP^Sc^ typing

**Fig. 2:**
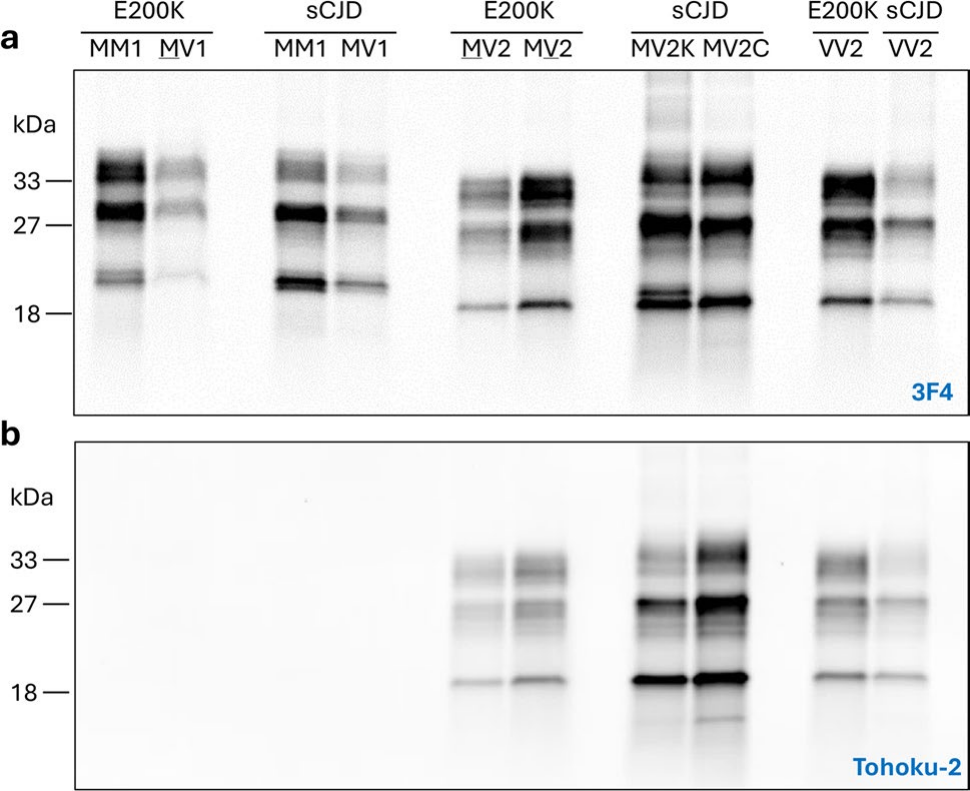
Typing of PK-resistant PrP^Sc^ in E200K and sCJD controls. Brain homogenates (S1) were treated with 10 U/ml PK. **a** and **b** The diglycosylated isoform of PrP^Sc^ is typically better represented in E200K than in sCJD. **a** The antibody 3F4 binds to T1 in E200K-MM(MV)1 and sCJDMM(MV)1 or T2 in E200K and sCJD -MV2, -VV2. Under the experimental conditions used in this study (see methods), the unglycosylated PrP^Sc^ T1 migrated to ~ 21–20 kDa, whereas that of T2 is  ~ 19 kDa. Unlike E200K-MV2, PrP^Sc^ of sCJDMV2K consists of the typical PrP^Sc^ doublet of ~ 20–19 kDa. **b** Tohoku-2 immunoreacts only with T2

We retrospectively examined the western blot profiles in 172 E200K cases. With the exception of one case, the di-glycosylated PrP^Sc^, and to a lesser extent, the mono-glycosylated isoform, were more represented than the un-glycosylated isoform [2, 27] (Fig. 2). One E200K-129MM case with overrepresentation of the mono-glycosylated PrP^Sc^ isoform showed an atypical histotype (not shown). Following immunoblotting of 18 E200K cases (see methods), both PrP^Sc^ types immunoreacted with 3F4 (Fig. 2a), whereas only T2 was labeled by Tohoku-2 (Fig. 2b). In E200K linked to MM(MV)1 subtype, 3F4 detected PrP^Sc^ T1. In E200K -MM(MV)2, -FI, -MV2 and -VV2, 3F4 and Tohoku-2 antibodies detected T2 only (Fig. 2 and Fig. S2). The amount of the PK-resistant PrP^Sc^ harvested from E200K -MM2 and -FI differed significantly. Unlike E200K-MM2, T2 of E200K-FI could be detected only after a tenfold enrichment of insoluble PrP^Sc^ (Fig. S2). A group of 14 E200K cases had mixed PrP^Sc^ types, with ratios of PrP^Sc^ types favoring (i) T1 in sCJDMM1-2 (n = 6), (ii) approximately both PrP^Sc^ types in MV1-2 (n = 7), and T2 in MV1-2 (n = 1).

### Clinical and histopathological characterization of E200K subtypes

The description of all E200K phenotypes and relative figures are shown in Table 2. Briefly, the *MM(MV)1* subtype is the most common as it accounts for 67% of the cases with -MM1 being significantly more represented than -MV1 (74% vs. 26%, P < 0.0001). All other subtypes were rarer and accounted for 7%-4% (i.e., MM(MV2), MV2, VV2, FI, MM1-2 and MV1-2) and < 1% (MV1-2) (Table 2).

**Fig. 3:**
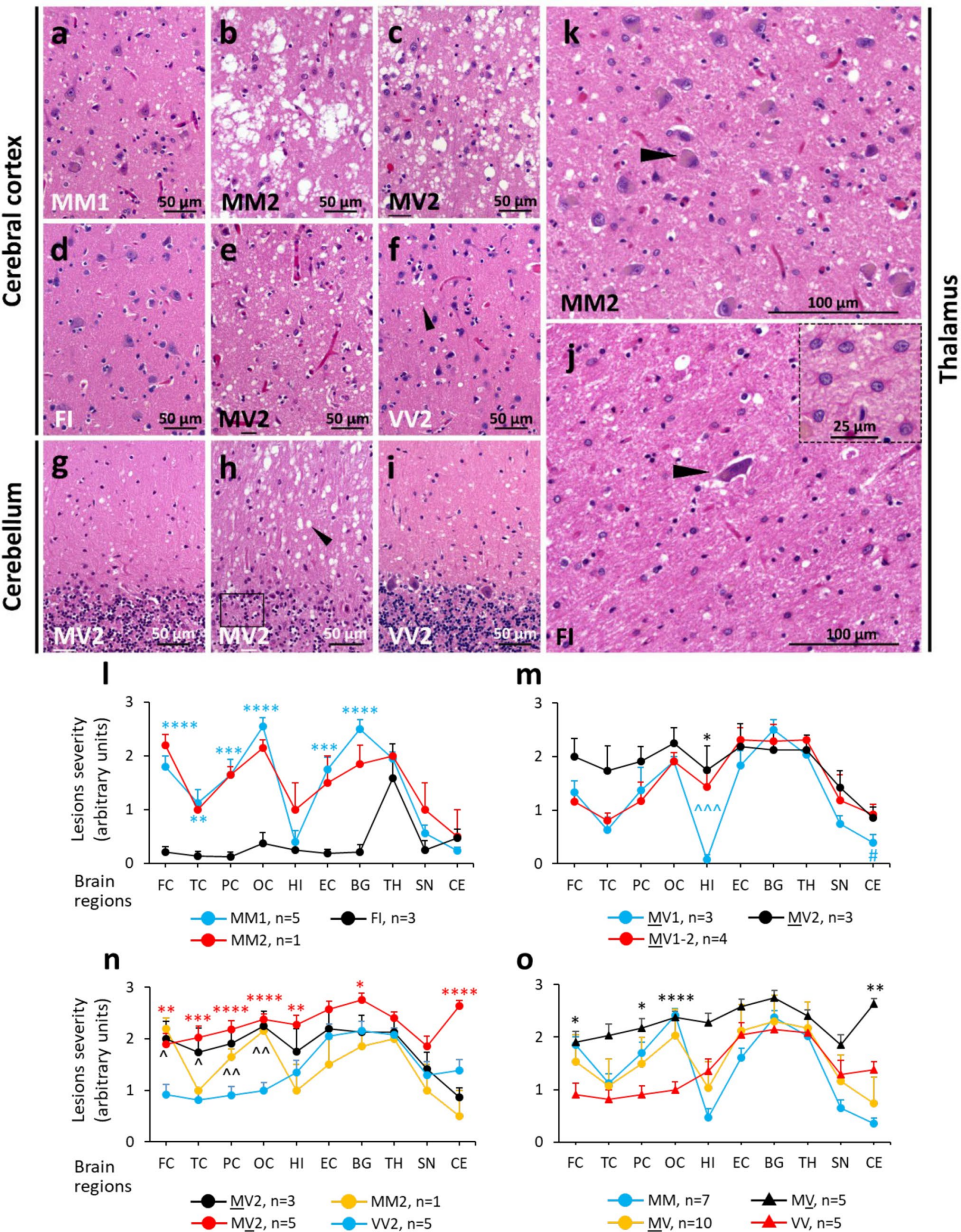
Histology and lesion profiles of E200K. **a**, **e**, **f**, **g**,** i** Small vacuoles spongiform degeneration (SD). **b** and **c** Large and confluent vacuoles. **d** No SD. **h** Medium-sized vacuoles (molecular layer) and cell depopulation (square, granular layer); arrowheads in **f** and **h** indicate small (**f**) and medium-sized (**h**) vacuoles. **j** and** k** Neuronal depopulation (**j**) and fairly normal number of neurons (**k**) of mediodorsal thalamic nuclei; inset, **j** reactive astrocytes; arrowhead in **j** and **k** indicate a neuron. **l** Lesion profile of FI differs significantly from those of MM1 and MM2 subtypes. **m** Unlike MV2, cortical lesions are less severe in MV1 and MV1-2; hippocampus is spared in MV1. **n** In VV2, cortical lesions are less severe than in MV2 and MM2. **o** Distinct lesion profiles of the 129M and 129V haplotypes. While lesion profiles of MM and MV genotypes are virtually identical, those of the MV and VV genotypes differ only in severity, but not in distribution. 129M haplotype: MM1 (n = 5), MM1-2 (n = 1), MM2 (n = 1); MV1 (n = 4), MV1-2 (n = 3), MV2 (n = 3). 129V haplotype: MV2 (n = 5), VV2 (n = 5). Student’s t-test compares MM1 vs. FI (*) (**l**), MV2 vs. MV1 (*), MV2 vs. MV1-2 (NS), MV1 vs. MV1-2 (^) (**m**), MV2 vs MV2 (NS), MV2 vs VV2 (^), MV2 vs VV2 (*) (**n**), MV vs VV (*); *P = 0.01–0.05; **P = 0.001–0.01; ***P = 0.0001–0.001; ****P < 0.0001

**Fig. 4:**
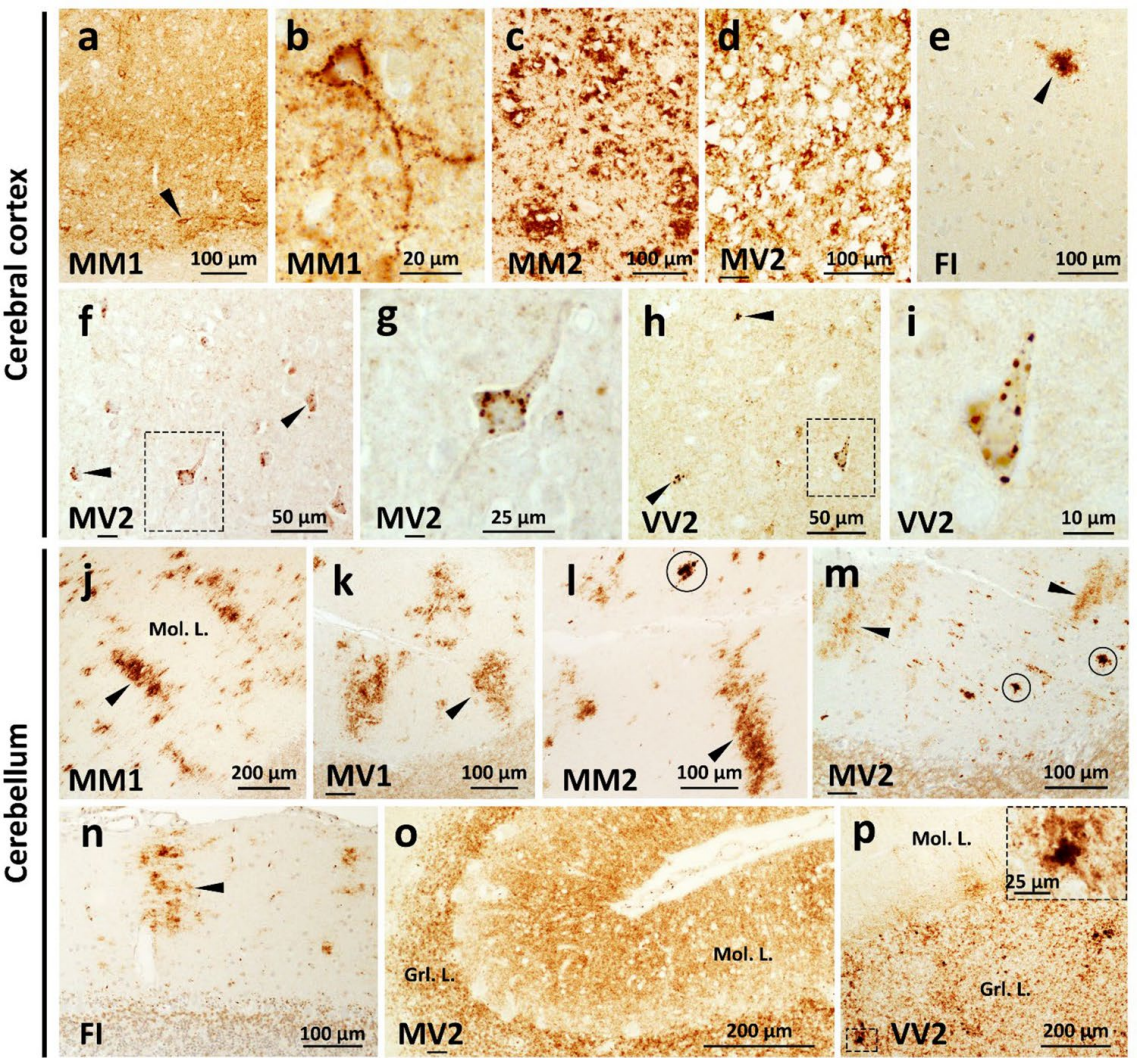
PrP immunohistochemistry. **a** Diffuse PrP immunostaining pattern; arrowheads: perineuronal PrP. **b** PrP deposits along nerve fiber tracts and neuronal perikaryon. **c** and **d** Coarse and perivacuolar PrP. **e**: A focus of coarse PrP (arrowhead). **f–i** Intracytoplasmic PrP deposits with a dot-like appearance; arrowheads in **f** and **h** indicate intraneuronal PrP deposits; **g** and **i** high magnification of neurons highlighted by the dashed rectangle in **f** and **h**. **j**–**n** Stripe-like PrP deposits (arrowheads); circles in **l** and **m** indicate coarse PrP. **o**: Diffuse PrP. **p** Plaque-like PrP; inset, **p** high magnification of a PrP plaque-like deposit. *Mol. L*. Molecular layer, *Grl. L*. Granular layer; antibody: 3F4

*Mixed PrP*^*Sc*^* types*. This group of 14 cases showed variable clinical features (Table 2). The histopathological phenotype resembled E200K-MM(MV)1 in most MM1-2 cases, MV1 and MV2 histotypic features in -MV1-2 cases, and MV2 in the -MV1-2 subtype (Tables 2 and S1) (Fig. 3, Fig. 4, and Fig. S3).

*Mixed phenotypical features of M and V haplotypes*. Four E200K-129MV harboring PrP^Sc^ T1 (n = 1) or both PrP^Sc^ types (n = 3) showed histotypic mixtures of the M and V haplotypes (Table 2). While clinical characteristics were variable, intraneuronal dot-like PrP immunostaining was found in all cases in addition to -MV1 and -MV1-2 histotypic features (Table 2 and Table S1) (Fig. 4).

### RT-QuIC

#### PrP seeding doses are comparable across E200K subtypes, but differ from those of fatal insomnia and sCJD

**Fig. 5:**
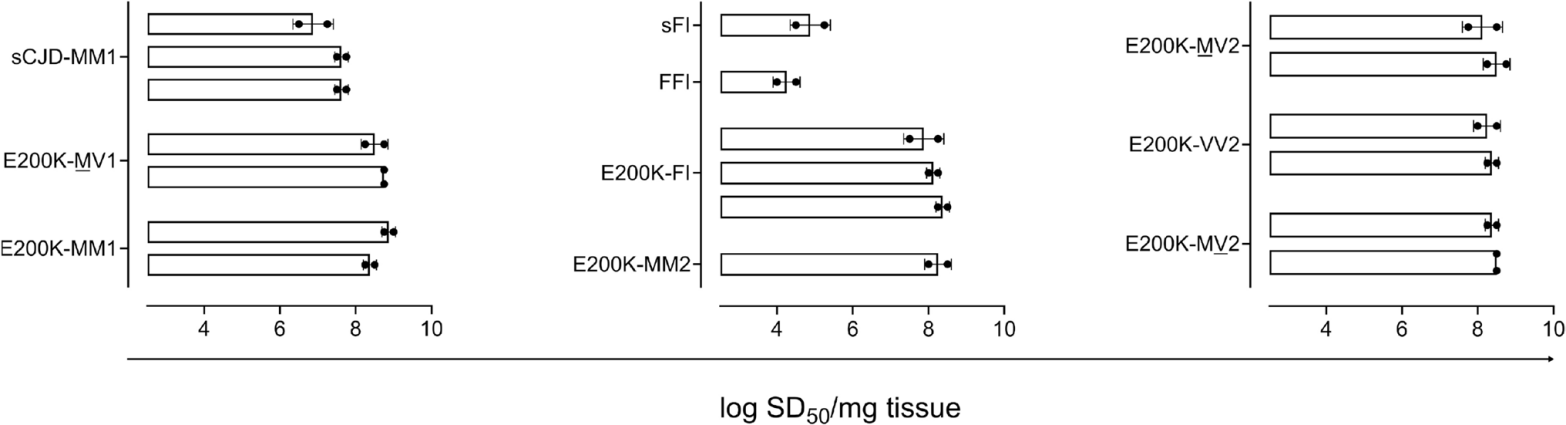
Endpoint-dilution RT-QuIC analysis. Seeding doses (log_10_SD_50_/mg tissue) were determined across E200K subtypes, with comparison to sCJD and fatal insomnia (FFI and sFI) cases. Each data point represents a technical replicate log10 SD_50_ value of an individual case. Each bar represents the mean log10 SD_50_ of an individual case (± SD)

Representative cases across E200K subtypes were evaluated using end-point dilution RT-QuIC analysis to estimate prion seeding doses in frontal cortex brain tissue homogenates. Across E200K cases, we detected 10^7^–10^9^ seeding doses per mg frontal cortex tissue (Fig. 5). This was ~ tenfold higher than seeding doses noted for sCJD cases (129MM, MM1 subtype) (MWU test, U = 0, P < 0.0002) and up to 10,000-fold higher than seeding activity assessed in a fatal familial insomnia and sporadic fatal insomnia case. ThT maximum curve amplitudes of E200K cases were lower than those noted for the sCJD cases (MWU test, U = 0, P < 003) (Fig. S4).

## Discussion

Although many *PRNP* mutations have been described, only a few are highly penetrant, with the most common being the E200K mutation [31, 35]. There are at least 4 ancestral clusters, including the two largest among Sephardic Jews and Slovakian cohorts. Other cohorts from Europe (Germany, Sicily, and Austria), and Japan have been reported [37, 39]. Most E200K cases are associated with 129 M haplotype and T1 PrP^Sc^ by western blot (albeit with predominance of the diglycosylated PrP^Sc^ isoform). Based on combined data from several published papers, most patients develop dementia (~ 95%) and cerebellar ataxia (~ 80%). Less common signs include extrapyramidal feature (~ 40%) and insomnia (~ 25%) [32, 33, 37].

In this study, we examined our collection of E200K cases to see if we could determine the effects of codon 129 polymorphisms on the disease phenotype. We also sought to investigate the relationships between these polymorphisms and the prion fragments recovered after PK digestion, as well as their effects on CSF and brain RT-QuIC analyses.

Nearly 90% of our cases were of the 129 M haplotype, three quarters of which were 129MM. Among these homozygotes, nearly 90% had PrP^Sc^ T1 only, with 5% showing PrP^Sc^ T2, and another 5% demonstrating both T1 and T2. Prion protein types showed greatest variability in our 40 patients with 129MV genotype. In this group, half showed PrP^Sc^ T1 only, while the other half was divided equally between both T1 and T2, and T2 only. Among the approximately 10% of patients expressing the 129 V haplotype (with just under half of these being homozygous), all but one heterozygote showed PrP^Sc^ T2 only.

As the complexity of mixed PrP^Sc^ type cases is still evolving, we decided to analyze our cases in the context of the original classification used for sporadic CJD. Like that classification, we found that patients with the 129 M haplotype and T1 could not be distinguished based on the polymorphism on the wild-type allele (although, as a group, heterozygotes had longer disease durations than homozygotes). Thus, our largest subtype, comprising two thirds of our cases, was E200K-MM(MV)1. These patients showed histopathological features similar to sCJDMM(MV)1, with fine spongiform degeneration involving cerebral cortex and subcortical gray matter with relative hippocampal and cerebellar sparing [46, 47]. This group also demonstrated the stripe-like immunostaining pattern in the cerebellar molecular layer, classically associated with the E200K mutation.

Patients with 129MM genotype and PrP^Sc^ T2 generated a FI phenotype (E200K-FI) [45] except for one case with cortical (C) coarse pathology mimicking sCJDMM2 (E200K-MM2C)[47]. Molecular features of E200K-MM2C were similar to those of sCJD, as they had comparable PrP^Sc^ glycotypes, with predominance of the mono-glycosylated PrP^Sc^ and high amounts of PK-resistant PrP^Sc^. Molecular features of E200K-FI resembled those of FI, and characterized by similar PrP^Sc^ glycotypes, with predominance of the di-glycosylated PrP^Sc^, and low amounts of PK-resistant PrP^Sc^, which required a tenfold pellet concentration (see methods). The fact that PrP^Sc^ T2, but not T1, shows a sCJD-like glycotype, emphasizes the properties of T1 and T2 as distinct isolates. In our cohort, the E200K-FI subtype was significantly more common than E200K-MM2C. This trend is in clear contrast with that seen in a recent study by Baiardi et al., where all E200K-129MM coupled with PrP^Sc^ T2 belonged to the E200K-MM2C subtype [2]. This discrepancy can perhaps be explained by the different E200K families populating these two cohorts. It should also be emphasized that none of the E200K-FI cases harbored PrP^Sc^ T1. Although this is what one would expect, the existence of E200K-FI in patients carrying PrP^Sc^ T1 cannot be excluded [55].

We have also observed in E200K patients with the 129 V haplotype comprising 10% of the entire E200K population, with half being homozygous (and virtually all associated with T2) ─ that 129MV and 129VV individuals showed distinctive intracytoplasmic dot-like immunoreactivity in the deep layers of the cerebral cortex. This intriguing phenotype deserves further study, as this immunoreactivity pattern suggests that the GPI-anchor is either absent or dysfunctional. As this intracytoplasmic dot-like PrP pattern is seen in all 129 V patients, methionine in the normal allele does not interfere with the formation of PrP aggregates. It is even possible that PrP^C^-129 M contributes to the dot-like PrP aggregation in these patients. Our (and Baiardi’s) data also indicate that dot-like PrP accumulates also in neurons of patients with the 129 M haplotype (hereafter referred as to E200K-129 M-dot-like), with the presence of valine in the normal allele potentially representing a prerequisite for this phenotype [2]. In our study, the majority of the E200K-129 M-dot-like cases showed a co-occurrence of PrP^Sc^ types. In Baiardi et al., the E200K-129 M-dot-like PK-resistant PrP^Sc^ migrated ~ 1 kDa faster than T1. However, our western blot analyses of E200K-129 M-dot-like (cases 14, 22–24 of Table S1) were run under experimental conditions dissimilar to those used by these authors [2]. 

In our study, four E200K-129 M-dot-like cases mimicked E200K-VV2 histopathology. However, the disease duration of our E200K-129 M-dot-like cases was 2.5 times longer than E200K-VV2 (P < 0.03) and E200K-129MV free of dot-like PrP deposits (P < 0.01).

Unlike this dot-like PrP pattern, perineuronal PrP was found in almost all E200K cases and did not signify any E200K subtype. Focal cerebellar plaque-like PrP was detected in two E200K -MM1, and three -MV1 cases, a histopathological feature not seen in sCJD of the MM(MV)1 subtype [6, 7, 13, 47].

We performed RT-QuIC on brain homogenates of E200K and controls. We found that seeding doses in frontal cortex did not differ across E200K subtypes. Frontal cortex E200K seeding activities were ~ tenfold higher than those noted for sCJD cases, and up to 10,000-fold higher than fatal insomnia. RT-QuIC seeding activity of E200K-FI was similar to other E200K subtypes, and significantly different from FFI/sFI controls. This suggests that despite its histopathological phenotype (i.e., very few vacuoles and scattered PrP staining in the cerebral cortex) and very low amount of PrP^Sc^ detected on western blot, the “prion strain” features of E200K-FI are driven by PrP^Sc^ itself as indicated by the short lag phase (Fig. 5 and S4).

E200K, sFI, and FFI RT-QuIC readouts had lower ThT fluorescence readouts when compared to the sCJD cases (n = 3) we examined in this study. Differences in ThT amplitudes for E200K seeded reactions when compared to sCJD or FFI have been reported in the literature, although the amplitudes, and whether E200K ThT curves are higher or lower, varies across the reports and by biospecimen type, substrate, and conditions used [36, 40, 51]. Regardless, differences in amplitudes are consistent with distinct sCJD, E200K, and FI prion strains. These findings also converge with a larger analysis of CSF specimens that detected the highest RT-QuIC positivity rate in E200K cases, followed by sCJD with variability between the different molecular subtypes, and no CSF RT-QuIC positive cases in FI [49].

Finally, our data show prion disease-positive MRI in the majority of E200K cases. The 70% sensitivity reported in this cohort is in line with the 75% found by Bizzi and coworkers in patients with gCJD [4]. The high sensitivity of MRI with diffusion imaging in detecting prion lesions in patients with E200K mutation is important as it confirms that MRI can be used to monitor prion lesions evolution during the natural course of the disease and to evaluate response to new therapies when they become available.

### Strengths/novelties and limitations of the study

*Scale and depth of phenotyping in E200K gCJD* This study represents the largest cohort of E200K cases analyzed to date, comprising 177 individuals with haplotype- and genotype-resolved *PRNP* codon 129 data. The comprehensive integration of clinical features, MRI and EEG findings, CSF biomarkers, detailed neuropathology, PrP^Sc^ typing by western blot, and RT-QuIC analyses provides an unusually deep multidimensional characterization of this common genetic prion disease.

*Expanded characterization of rare cis-129 V and 129VV genotypes* The cohort includes 21 cases in which the E200K mutation occurs on the cis-129 V haplotype, including nine 129VV cases. This allowed a more detailed analysis of these rare genotypes, demonstrating a strong association with PrP^Sc^ T2 predominance and distinctive histopathological features. Given that most published E200K series are heavily dominated by cis-129 M cases, this represents a meaningful incremental advance.

*Refinement of the FI-like E200K phenotype and its molecular context* We further delineate an atypical fatal insomnia–like (FI-like) E200K subtype and contrast it with E200K-MM2C. Although FI-like E200K has been reported previously, our study provides new insight by integrating cohort prevalence, biochemical features, and RT-QuIC data. Notably, FI-like cases showed very low levels of PK-resistant PrP^Sc^ by conventional western blot, requiring enrichment for detection, yet exhibited robust prion seeding activity by RT-QuIC. This combination supports the notion that subtle biochemical properties, rather than absolute PrP^Sc^ burden, underlie this phenotype.

*Quantitative brain RT-QuIC endpoint-dilution analysis across E200K subtypes* While most studies focus on RT-QuIC sensitivity or positivity our endpoint-dilution analysis provides quantitative insight into prion seeding activity across genetic subtypes. Specifically, we demonstrate comparable seeding doses among E200K subtypes, with levels markedly higher than those observed in FI, together with distinct differences in ThT fluorescence kinetics. These data support the concept of strain-related differences within genetic prion disease and add novel quantitative information on E200K-associated prion biology. At the same time, the immediate diagnostic or translational impact of these differences is currently limited, given the reliance on *post-mortem* brain tissue and the difficulty of reliably determining prion subtypes in life. Accordingly, our findings should be seen as primarily informative for prion biology and strain characterization, with potential translational relevance contingent on future advances in in-vivo subtype discrimination.

Despite these strengths, several limitations should be acknowledged. *First*, the study is retrospective and relies on surveillance and brain-bank data, resulting in incomplete availability of certain clinical, imaging, biomarker, and molecular variables. Consequently, some analyses were performed on reduced sample sizes, particularly for advanced molecular or imaging endpoints. *Second*, despite the large overall cohort, several clinically and biologically important subgroups remain numerically small, including cis-129 V haplotype cases, VV2, FI-like, MM2, and mixed PrP^Sc^ type groups. Statistical comparisons involving these subtypes may therefore be underpowered and sensitive to individual cases. *Third*, all cases were ascertained through a single national reference center (NPDPSC). Differences in referral patterns, diagnostic practices, and population ancestry may limit generalizability to other E200K populations with distinct founder effects, such as Slovakian, Sephardic, or East Asian cohorts. *Fourth*, PrP^Sc^ typing was performed in a limited number of brain regions. Because mixed PrP^Sc^ types are known to exhibit regional variability, this approach may underestimate their true prevalence, and some cases classified as “pure” T1 or T2 may harbor minor secondary PrP^Sc^ components not detected under the sampling strategy used. *Finally*, quantitative endpoint-dilution RT-QuIC analyses were performed on selected representative cases rather than the full cohort. Although these results provide valuable comparative insights, they should be interpreted as exploratory and require confirmation in larger, systematically sampled series.

## Conclusions

The present study describes the largest cohort of E200K-associated gCJD to date and highlights the clinicopathologic heterogeneity associated with this mutation. We identify five histopathologically distinct E200K subtypes, of which MM(MV)1 is the most prevalent (~ 70%). The E200K -MM(MV)2, -FI, -MV2 and -VV2 are less common but readily recognizable.

As observed in other human prion diseases, PrP^Sc^ type distribution, clinical manifestations, and neuropathological phenotypes are strongly influenced by the codon 129 polymorphism. The atypical E200K-FI phenotype and its divergence from E200K-MM2C is likely to be driven by subtle molecular/biochemical features [12]. Further studies integrating multi-omics approaches and high-resolution structural analyses, including cryo-electron microscopy will be essential to elucidate the mechanisms underlying the emergence of fatal-insomnia–like phenotypes in E200K carriers.

## Supplementary Information

Below is the link to the electronic supplementary material.

Supplementary file1 (JPG 230 KB)

Supplementary file2 (JPG 459 KB)

Supplementary file3 (JPG 2568 KB)

Supplementary file4 (JPG 424 KB)

Supplementary file5 (DOCX 26 KB)

Supplementary file6 (DOCX 31 KB)
