# Supplementary figures and images for: Genetic Creutzfeldt-Jakob disease linked to the E200K mutation: a large cohort study
Source: Acta Neuropathol. 2026 Jan 13;151(1):5. doi: 10.1007/s00401-026-02975-x (PMC12799623; doi:10.1007/s00401-026-02975-x)

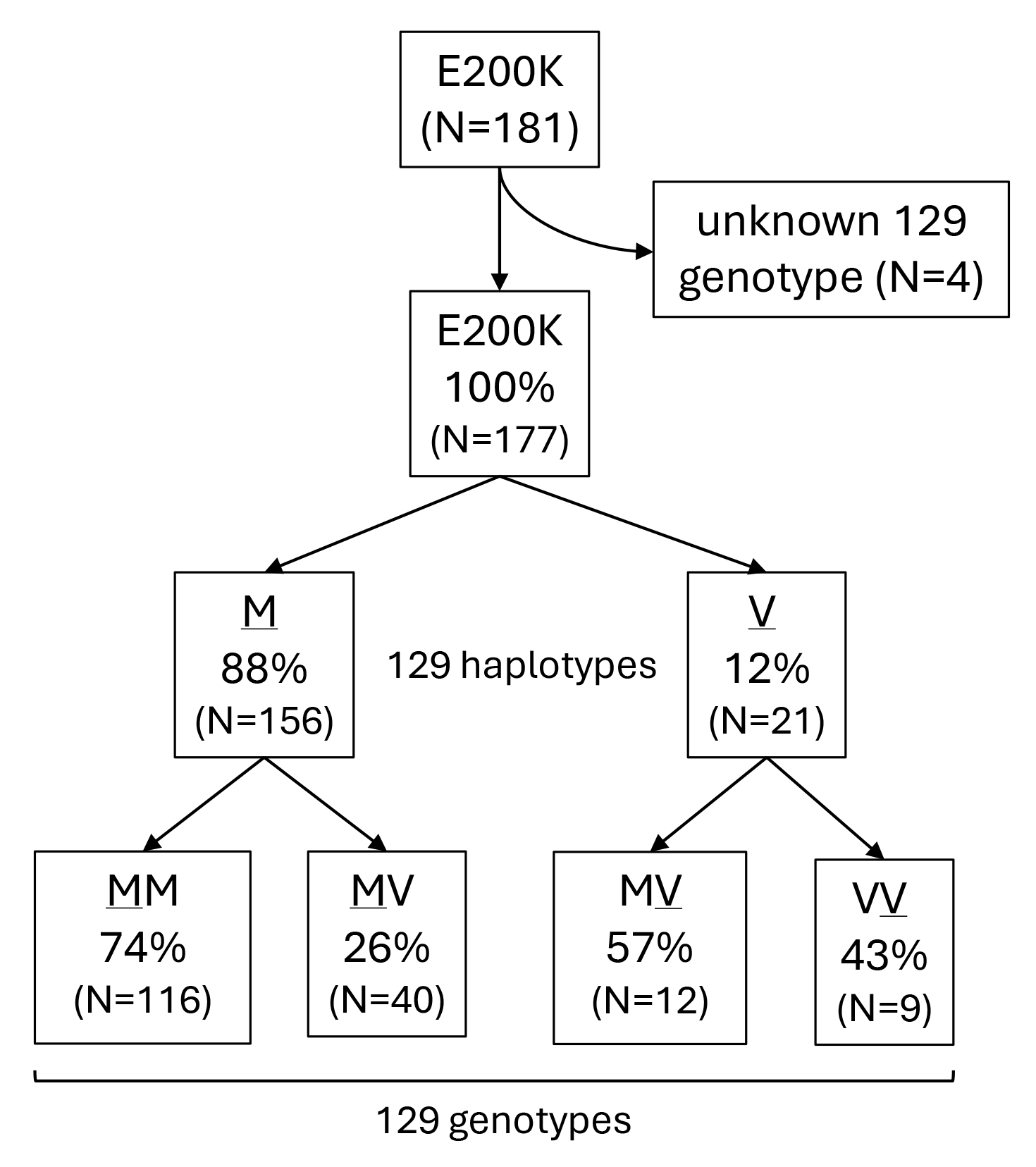

Supplement: Supplementary file 1 — Supplementary file1 (JPG 230 KB) [file 401_2026_2975_MOESM1_ESM.jpg]

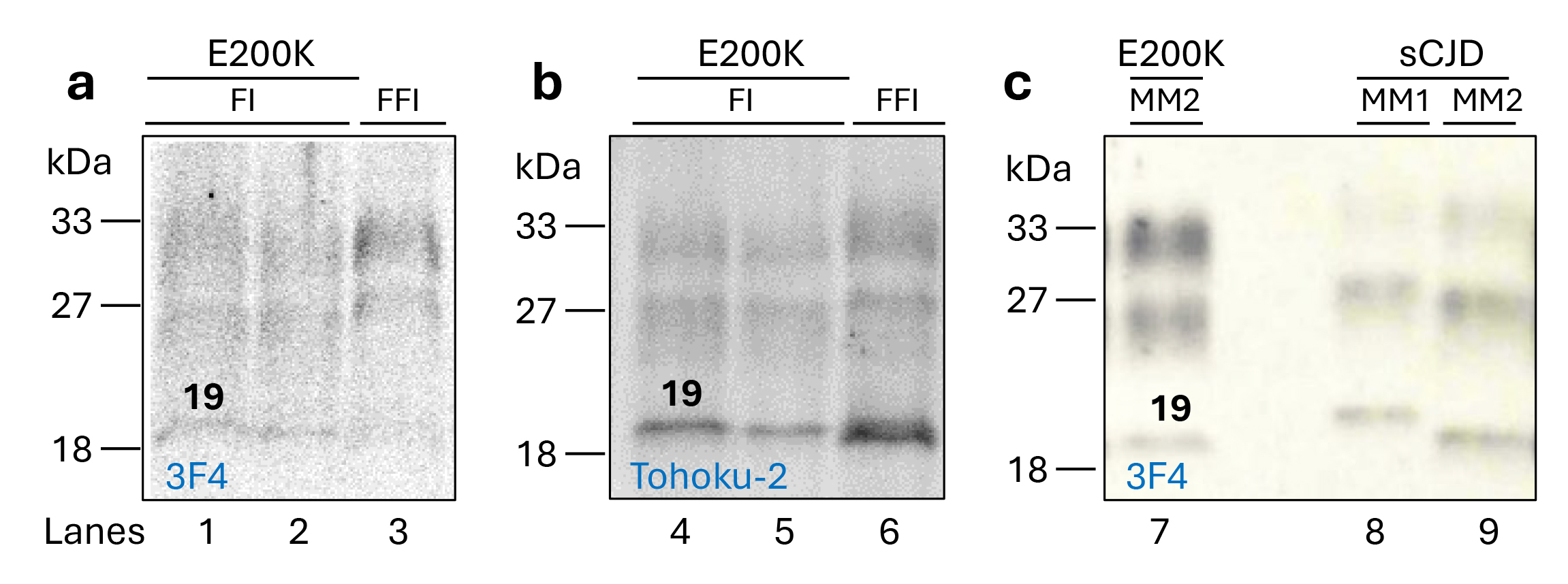

Supplement: Supplementary file 2 — Supplementary file2 (JPG 459 KB) [file 401_2026_2975_MOESM2_ESM.jpg]

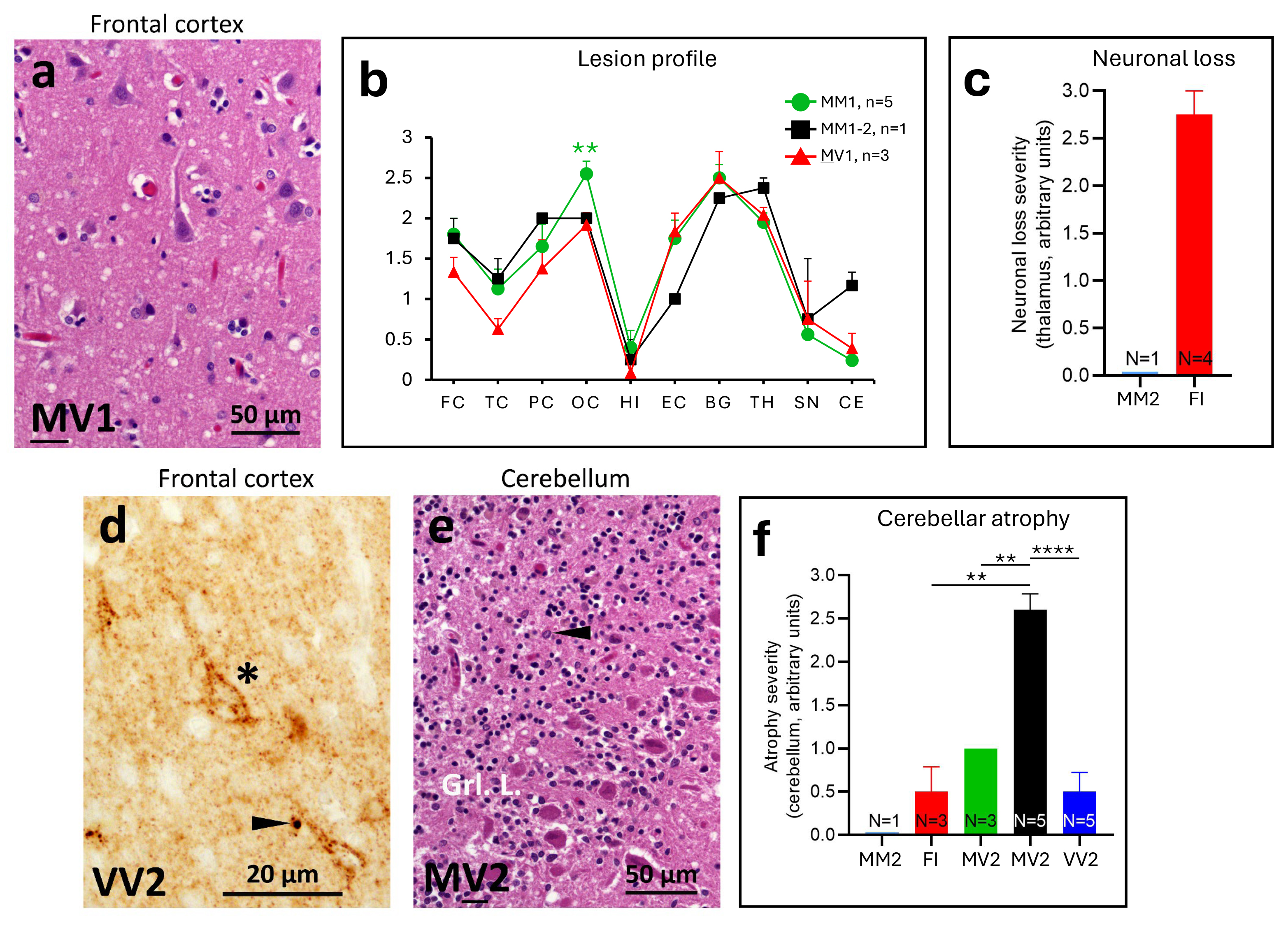

Supplement: Supplementary file 3 — Supplementary file3 (JPG 2568 KB) [file 401_2026_2975_MOESM3_ESM.jpg]

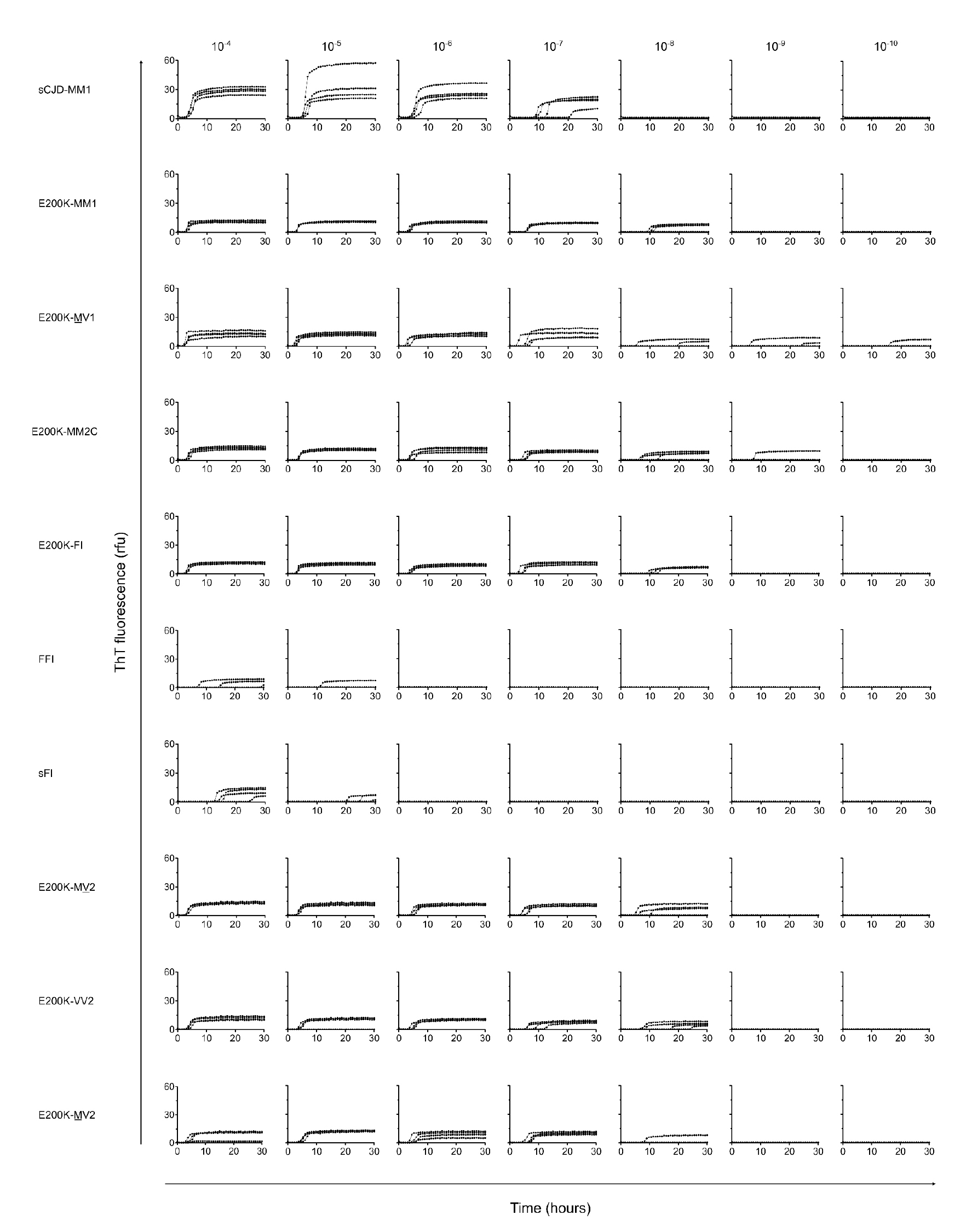

Supplement: Supplementary file 4 — Supplementary file4 (JPG 424 KB) [file 401_2026_2975_MOESM4_ESM.jpg]
