# Supplementary material for: Genetic Creutzfeldt-Jakob disease linked to the E200K mutation: a large cohort study
Source: Acta Neuropathol. 2026 Jan 13;151(1):5. doi: 10.1007/s00401-026-02975-x (PMC12799623; doi:10.1007/s00401-026-02975-x)
Supplement: Supplementary file 5 — Supplementary file5 (DOCX 26 KB) [file 401_2026_2975_MOESM5_ESM.docx]

**Genetic Creutzfeldt-Jakob disease linked to the E200K mutation: a large cohort study**

Brian S. Appleby,^1,2,3,4^ Matteo Manca,^1^ Megan S. Piazza,^5,6^ Travis D. Kerr,^7^ Antonio Cornacchia,^1^ Alberto Bizzi,^8^ Allison Kraus,^1,4^ Mark L. Cohen,^1,4^ Ignazio Cali ^1,4,7^

Departments of ^1^Pathology, ^2^Neurology, and ^3^Psychiatry, ^5^Center for Human Genetics Laboratory, School of Medicine, Case Western Reserve University; ^6^University Hospitals Cleveland Medical Center, ^4^National Prion Disease Pathology Surveillance Center, Cleveland, OH 44106, USA; ^7^Center for Immunotherapy and Precision Immuno-Oncology, Lerner Research Institute, Cleveland Clinic, Cleveland, OH, USA; ^8^UOC Neuroradiologia, Fondazione IRCCS Istituto Neurologico Carlo Besta, 20133 Milan, Italy; ^7^Kore University, Department of Medicine and Surgery, Enna, EN 94100, Italy.

Corresponding authors: [bsa35@case.edu](mailto:bsa35@case.edu) and [ixc20@case.edu](mailto:ixc20@case.edu)

Tel: 216-368-0587

Fax: 216-368-4090

**Figure legends**

Fig. S1 **Schematic representation of the E200K cohort**. Percentage (%) and number (N) of cases associated with 129 -M and -V haplotypes and 129 -MM, -MV and -VV genotypes are indicated within each box.

Fig. S2 **PrP^Sc^ western blot profile of E200K MM2 and FI subtypes**. Insoluble PrP^Sc^ fractions (P2) (**a** and **b**) and S1 brain homogenates (**c**) were treated with 5 U/ml (**a** and **b**) and 10 U/ml (**c**) PK. **a**: The insoluble unglycosylated PrP^Sc^ isoform of two E200K FI cases (lanes 1 and 2) and one FFI (lane 3) migrated to ~19 kDa (T2). Unlike 3F4 (**a**), the unglycosylated PrP^Sc^ of E200K FI (lane 4 and 5) and FFI (lane 6) appears more intense than mono- and diglycosylated isoforms when probed with Tohoku-2. **c**: Unglycosylated PrP^Sc^ isoform of E200K MM2 (lane 7) migrated to ~19 kDa, matching the gel mobility of sCJDMM2 PrP^Sc^ (lane 9); lane 8: sCJDMM1 control.

Fig. S3 **Histopathology of E200K. a** and **e**: Hematoxylin-eosin staining. **d**: PrP immunohistochemistry. **a:** Fine spongiform degeneration. **b**: Similar lesion profiles of MM1, MM1-2 and MV1 (MM1 vs. MV1, **P<0.02; Student’s t-test). **c**: Severe loss of thalamic nuclei in FI, but not MM2. **d**: Granular deposits distributed around the neuron perikaryon (asterisk); arrowhead: a larger PrP granule; antibody: 3F4. **e**: Loss of granule cells and gliosis; arrowhead: an astrocyte. **f**: Cerebellar atrophy is most severe in E200K-MV2; **P=0.001-0.01; ****P<0.0001; one-way ANOVA).

Fig. S4. **Endpoint dilution RT-QuIC analysis**. Normalized ThT fluorescence curves of representative cases are shown for quadruplicate well analysis of indicated brain homogenate dilutions (10^-4^ – 10^-10^) to calculate seeding doses shown in Fig. 5. Rfu = normalized relative fluorescence units.
