# Supplementary material for: Genetic Creutzfeldt-Jakob disease linked to the E200K mutation: a large cohort study
Source: Acta Neuropathol. 2026 Jan 13;151(1):5. doi: 10.1007/s00401-026-02975-x (PMC12799623; doi:10.1007/s00401-026-02975-x)
Supplement: Supplementary file 6 — Supplementary file6 (DOCX 31 KB) [file 401_2026_2975_MOESM6_ESM.docx]

| **Table S1**. Histopathological features of E200K | | | | | | | | | | | | | | | |
| --- | --- | --- | --- | --- | --- | --- | --- | --- | --- | --- | --- | --- | --- | --- | --- |
| E200K subtype | Case number | Age at death (years) | | Disease durat. (months) | | Cerebral Cortex (CC)^a^ | | | |  | Cerebellum | | | | |
|  |  |  |  |  |  | Histology |  | PrP immunohistochemistry | |  | Histology | |  | PrP immunohistochemistry | |
|  |  |  |  |  |  | Large vacuoles |  | Intra-neuronal,  dot-like | % surface CC with coarse PrP |  | Large vacuoles | Loss of  granule cells |  | Stripe-  like,  Mol. L. | Plaque-like,  Grl. L. |
| MM1 | 1 | 63 | | 5 | | – |  | – | 0 |  | – | 0 |  | + | – |
|  | 2 | 62 | | 2 | | – |  | – | 0 |  | – | 0 |  | + | + |
|  | 3 | 62 | | 3 | | – |  | – | 0 |  | – | 0 |  | + | + |
|  | 4 | 60 | | 3 | | – |  | – | 0 |  | – | 0 |  | + | – |
|  | 5 | 58 | | 5 | | – |  | – | 0 |  | – | 0 |  | + | – |
|  | | | 61±2^b^ | | 4±1^b^ | 0^c^ |  | 0^c^ | 0^b^ |  | 0^c^ | 0 |  | 100^c^ | 40^c^ |
| FI | 6 | 46 | | 3 | | – |  | – | 1 |  | – | 0.5 |  | + | – |
|  | 7 | 59 | | 2 | | – |  | – | 0 |  | – | 1 |  | + | – |
|  | 8 | 67 | | 3 | | – |  | – | 1 |  | – | 0 |  | + | – |
|  | | | 57±11^b^ | | 3±0.6^b^ | 0^c^ |  | 0^c^ | 0.7±0.6^b^ |  | 0^c^ | 0.5±0.5^b^ |  | 100^c^ | 0^c^ |
| *FI vs MM1^d^* | | | *NS* | | *NS* | *NS* |  | *NS* | *NS^e^* |  | *NS* | *NS^e^* |  | *NS* | *NS* |
| MM2 | 9 | 77 | | 13 | | + |  | – | 70 |  | – | – |  | + | – |
| **MV1** | 10 | 60 | | 2 | | – |  | – | 0 |  | – | 0 |  | + | + |
|  | 11 | 54 | | 3 | | – |  | – | 0 |  | – | 0 |  | + | + |
|  | 12 | 62 | | 5 | | – |  | – | 0 |  | – | 0 |  | + | – |
|  | 13 | 74 | | 2 | | – |  | – | 0 |  | – | 0 |  | + | – |
|  | 14 | 62 | | 9 | | – |  | + | 0 |  | – | 0 |  | + ^f^ | – |
|  | 15 | 55 | | 3 | | – |  | – | 0 |  | – | 0 |  | + ^f^ | + |
|  | 16 | 74 | | 4 | | – |  | – | 0 |  | – | 0 |  | + | – |
|  | | | 63±8^b^ | | 4±2^b^ | 0^c^ |  | 14^c^ | 0^b^ |  | 0^c^ | 0 |  | 100^c^ | 43^c^ |
| *MV1 vs MM1^d^* | | | *NS* | | *NS* | *NS* |  | *NS* | *NS^e^* |  | *NS* | *NS^e^* |  | *NS* | *NS* |
| **MV1-2** | 17 | 77 | | 3 | | + |  | – | 20 |  | – | 0 |  | + | + |
|  | 18 | 60 | | 7 | | + |  | – | 75 |  | – | 0 |  | + | – |
|  | 19 | 64 | | 9 | | + |  | – | 5 |  | – | 0.5 |  | + | + |
|  | 20 | 65 | | 11 | | + |  | – | 65 |  | – | 1.5 |  | + | – |
|  | 21 | 75 | | 4 | | + |  | – | 65 |  | – | 0.5 |  | + ^f^ | – |
|  | 22 | 63 | | 8 | | – |  | + | 0 |  | – | 0 |  | + | – |
|  | 23 | 74 | | 29 | | + |  | + | 20 |  | – | 0 |  | + ^f^ | – |
|  | 24 | 63 | | 11 | | + |  | + | 20 |  | – | 0 |  | + ^f^ | – |
|  | | | 68±7^b^ | | 10±8^b^ | 88^c^ |  | 38^c^ | 34±30^b^ |  | 0^c^ | 0.3±0.5^b^ |  | 100^c^ | 25^c^ |
| *MV1-2 vs MV1^d^* | | | NS | | NS | <0.002 |  | NS | <0.02^e^ |  | NS | NS^e^ |  | NS | NS |
| **MV2** | 25 | 73 | | 18 | | + |  | – | 45 |  | – | 1 |  | + | + |
|  | 26 | 67 | | 28 | | + |  | – | 100 |  | – | 1 |  | + | + |
|  | 27 | 64 | | 32 | | + |  | – | 100 |  | – | 1 |  | + | – |
|  | | | 68±5^b^ | | 26±7^b^ | 100^c^ |  | 0^c^ | 82±32^b^ |  | 0^c^ | 1 |  | 100^c^ | 67^c^ |
| *MV2 vs MV1-2^d^* | | | *NS* | | *<0.006* | *NS* |  | *NS* | *NS^e^* |  | *NS* | *NS^e^* |  | *NS* | *NS* |
| **MV2** | 28 | 55 | | 7 | | – |  | + | 0 |  | + | 3 |  | – | – |
|  | 29 | 66 | | 7 | | – |  | + | 0 |  | + | 2 |  | – | – |
|  | 30 | 67 | | 8 | | – |  | + | 1 |  | + | 2.5 |  | – | – |
|  | 31 | 58 | | 7 | | – |  | + | 0 |  | + | 3 |  | – | – |
|  | 32 | 48 | | 8 | | – |  | + | 0 |  | + | 2.5 |  | – | – |
|  | | | 59±8^b^ | | 7±0.5^b^ | 0^c^ |  | 100^c^ | 0.2±0.5^b^ |  | 100^c^ | 3±0.5^b^ |  | 0^c^ | 0^c^ |
| *MV2 vs MV2^d^* | | | NS | | <0.05 | <0.02 |  | <0.02 | <0.05^e^ |  | <0.02 | <0.002^e^ |  | <0.02 | NS |
| **VV2** | 33 | 55 | | 2 | | – |  | + | 0 |  | – | 1 |  | – | + |
|  | 34 | 46 | | 4 | | – |  | + | 0 |  | – | 0 |  | – | + |
|  | 35 | 71 | | 2 | | – |  | + | 0 |  | – | 0.5 |  | – | + |
|  | 36 | 59 | | 2 | | – |  | + | 0 |  | – | 1 |  | – | + |
|  | 37 | 66 | | 3 | | – |  | + | 0 |  | – | 0 |  | – | + |
|  | | | 59±10^b^ | | 3±0.9^b^ | 0^c^ |  | 100^c^ | 0 |  | 0^c^ | 0.4±0.5^b^ |  | 0^c^ | 100^c^ |
| *VV2 vs MV2^d^* | | | *NS* | | *<0.0001* | *NS* |  | *NS* | *NS^e^* |  | *<0.008* | *<0.0001^e^* |  | *NS* | *<0.008* |
| ^a^ Averaged values of frontal, temporal, parietal, occipital and entorhinal cortices. ^b,c^ Expressed as ^b^ mean±SD and ^c^ percentage; ^d^ Fisher’s exact test; ^e^ Student’s t-test. ^f^ Mix of diffuse and stripe-like PrP patterns. NS: not significant. Mol. L.: Molecular layer; Grl. L.: Granular layer. | | | | | | | | | | | | | | | |
